# Influence of CurQfen^®^-curcumin on cognitive impairment: a randomized, double-blinded, placebo-controlled, 3-arm, 3-sequence comparative study

**DOI:** 10.3389/frdem.2023.1222708

**Published:** 2023-09-13

**Authors:** S. Syam Das, Prasad M. Gopal, Jestin V. Thomas, Mohind C. Mohan, Siju C. Thomas, Balu P. Maliakel, I. M. Krishnakumar, Baby Chakrapani Pulikkaparambil Sasidharan

**Affiliations:** ^1^Akay Natural Ingredients, Kochi, Kerala, India; ^2^Alzheimer's and Related Disorders Society of India, Kochi, Kerala, India; ^3^Centre for Neuroscience, Cochin University of Science and Technology, Kochi, Kerala, India; ^4^Leads Clinical Research & Bio Services Private Limited, Bengaluru, India; ^5^Department of Biotechnology, Cochin University of Science and Technology, Kochi, Kerala, India; ^6^Centre for Excellence in Neurodegeneration and Brain Health, Kochi, Kerala, India

**Keywords:** Alzheimer's disease, bioavailability, blood-brain-barrier, CGM, clinical trial, curcumin, dementia

## Abstract

**Background:**

Although curcumin is a blood-brain-barrier permeable molecule with the ability to bind and segregate β-amyloid plaques and neurofibrillary tangles of hyperphosphorylated tau proteins, its poor oral bioavailability, rapid biotransformation to inactive metabolites, fast elimination from the systemic circulation, and hence the poor neuronal uptake has been limiting its clinical efficacy under neurodegenerative conditions.

**Objective:**

We hypothesized that the highly bioavailable CurQfen-curcumin (CGM), which has been shown to possess significant blood-brain-barrier permeability and brain bioavailability, would ameliorate dementia in neurodegenerative conditions.

**Methods:**

In the present double-blinded placebo-controlled 3-arm 3-sequence comparative study, 48 subjects characterized with moderate dementia due to the onset of Alzheimer's disease were randomized into three groups (*N* = 16/group) and supplemented with 400 mg × 2/day of either placebo (MCC), unformulated standard curcumin complex with 95% purity (USC), or CGM as a sachet for six months. The relative changes in cognitive and locomotor functions and biochemical markers were compared.

**Results:**

Supplementation with CGM produced significant (*P* < 0.05) improvement in the Mini-Mental State Examination (MMSE) and the Geriatric Locomotive Function Scale (GLFS) scores in both intra- and inter-group comparison by 2 × 2 repeated measures (RM) ANOVA. Further, analysis of the serum levels of specific biomarkers (BDNF, Aβ42, tau protein, IL-6, and TNF-α) also revealed a significant (*P* < 0.05) improvement among CGM subjects as compared to placebo and the USC groups.

**Conclusion:**

Supplementation with CGM as sachet was found to offer significant delay in the progress of Alzheimer's disease, as evident from the improvements in locomotive and cognitive functions related to dementia.

**Clinical trial registration:**

http://ctri.nic.in, identifier: CTRI/2018/03/012410.

## 1. Introduction

Dementia is a syndrome involving the progressive deterioration of cognition (Arevalo-Rodriguez et al., 2015). More than 50 million people worldwide are believed to suffer from dementia-related issues such as dependence, poor quality of life, and institutionalization (Prince et al., 2015; Lynch, 2020). Dementia cases are expected to reach 152 million by 2050 (Nichols et al., 2022). Approximately 8.4 million Americans over the age of 65 are expected to be affected by 2030, at a healthcare cost of more than USD$ 400 billion (Gooch et al., 2017; 2021). Though older populations are more often prone to dementia complications, recent studies revealed that younger individuals may also be affected; symptoms may only be visible in the later phases (Moffitt et al., 2017; Kvello-Alme et al., 2019; Elliott et al., 2021). Sedentary lifestyle, smoking, alcoholism, and stress have been correlated with the early onset of dementia (Llewellyn et al., 2019; Franks et al., 2021). COVID-19 pandemic-imposed quarantine and subsequent lifestyle changes have also been reported to significantly contribute to the increased risk of dementia (di Santo et al., 2020).

More than 70% of all dementia cases are attributed to Alzheimer's disease (AD), a complex disease involving significant changes in genetic and chemical makeup with the deregulation of protein synthesis (James and Bennett, 2019). The pathophysiology of AD is associated with elevated abnormal phosphorylation of tau protein, leading to the formation of neurofibrillary tangles in the brain (Arriagada et al., 1992). Along with the tau phosphorylation, the aggregation and accumulation of amyloid β (Aβ42 and Aβ40) also play a key role. A higher ratio of Aβ42/Aβ40 in the blood is very critical for the early onset of AD and hence neurotoxicity (Kumar-Singh et al., 2006; Kuperstein et al., 2010). Studies have shown that the level of Aβ42/Aβ40 decreases in cerebrospinal fluid (CSF), while the blood ratio increases with the pathogenesis of dementia (Teunissen et al., 2018). Brain-derived neurotrophic factor (BDNF), one of the neurotrophic factors found in the memory-related brain areas (hippocampus and amygdala), is also implicated in cognition and neuronal survival. Low levels or lack of BDNF have been shown to be linked to the loss of memory in various neurodegenerative conditions and age-related cognitive deficits (Nagahara et al., 2009; Miranda et al., 2019). Clinical trials have shown a decreased serum BDNF in neurological disorders, signifying BDNF as a potential biomarker (Diniz and Teixeira, 2011; Jiao et al., 2016). Multiple studies have observed a correlation among blood BDNF, frontal cortex and hippocampal BDNF, suggesting that blood or plasma BDNF reflects brain tissue BDNF levels (Klein et al., 2011). Another factor contributing to dementia is neuroinflammation. Higher expression of C-reactive protein (CRP), proinflammatory cytokines IL-1β, IL-6, and TNF-α, and chitinase-3 like protein I (CHI3L1 or YKL-40) have been reported to be associated with the pathogenesis of AD (Wang et al., 2015; Kim et al., 2017; Shen et al., 2019).

There is still a lack of effective therapeutic strategies and clinical practices for the cure of AD. The current treatment mainly includes cholinesterase inhibitors for mild AD and memantine treatment for moderate AD conditions, neither of which alters the course of the disease or the rate of cognitive decline. The newly introduced disease-modifying biological drug, aducanumab, only acts to clear Aβ aggregates and has no clinical benefits in clearing Aβ aggregates in persons with sporadic AD (Perlmutter, 2021). Side-effects and the cost/dose of available drugs also constitute a major concern with the majority of the dementia population.

There has recently been a move toward natural remedies for improving brain health and especially, for improving or maintaining cognitive and locomotive functions. Curcumin, the yellow pigment in turmeric, has shown significant anti-neuro-inflammatory effects and the ability to stimulate hippocampal neurogenesis and synaptogenesis (Gagliardi et al., 2020; Yi et al., 2020). The plausible efficacy of curcumin has been reported in hippocampus dysfunction mediated by conditions of stroke, trauma, radiation, and neurodegenerative diseases (Benameur et al., 2021). However, it has failed to provide significant clinical efficacy in neurodegenerative conditions. Poor oral bioavailability, metabolism to inactive metabolites, rapid systemic elimination, and negligible brain tissue distribution (brain bioavailability) have been identified as its main limitations (Krishnakumar et al., 2015; Gagliardi et al., 2020). When consumed, it is only poorly absorbed (<1%) due to insolubility and incompatibility with gastrointestinal conditions, and the absorbed fraction undergoes rapid glucuronidation/sulfation to inactive metabolites with poor cellular permeability and rapid elimination from systemic circulation (Gagliardi et al., 2020).

Curcumin-galactomannan complex (CGM) is a self-emulsifying hydrogel delivery system developed by the uniform encapsulation of curcumin within the fenugreek-derived galactomannan (soluble dietary fiber) hydrogel matrix. It has been shown to possess enhanced bioavailability of free (unconjugated) curcuminoids, improved blood–brain-barrier (BBB) permeability, brain bioavailability, and improved neuroprotective effects compared to unformulated standard curcumin complex with 95% purity (USC) (Krishnakumar et al., 2015; Kumar et al., 2016; Khanna et al., 2020). We hypothesized that CGM would be able to ameliorate the conditions of Alzheimer's disease upon continuous supplementation for longer duration. Thus, the present study was conducted on subjects having normal hematological and biochemical serum parameters, but characterized with moderate dementia due to the onset of AD as evident from their Mini-Mental State Exam (MMSE) score. A randomized, double-blinded, placebo-controlled, 3-arm, 3-sequence, comparative study was executed with the supplementation of 400 mg × 2/day of either placebo (microcrystalline cellulose, MCC), an unformulated standard curcumin complex with 95% purity (USC) or CGM as a sachet for 6 months. Considering the general difficulty that dementia subjects experience in swallowing capsules/tablets, the present study used sachets (a 3 g sachet containing 400 mg of the test substance to be consumed by mixing with water).

## 2. Materials and methods

### 2.1. Test substances

Unformulated standard curcumin complex with >95% purity (USC) was extracted from dried Cambodian turmeric (*Curcuma Longa L*), which has a curcumin content of 10.8%, using ethyl acetate extraction. The proprietary formulation of USC with fenugreek galactomannan dietary fiber is a water-dispersible powder (curcumin-galactomannan complex, CGM). Identical sachets of USC, MCC (placebo), and CGM were provided in identical bottles (65 sachets per bottle). Each sachet weighed ~3 g with 400 mg of the test substance and the remaining maltodextrin with <10 dextrose equivalents. The placebo (MCC) was colored with the approved food color, sunset yellow, to look like curcumin and flavored with 250 ppm turmeric oil to match the sensory aspects. Stevia was used as a low-calorie sweetener for the formulation. A detailed certificate of analysis confirming its food-grade status along with the respective composition statement and material safety data sheet were obtained from the manufacturer. The purity and composition of curcuminoids in sachets were determined by the validated USP method (Jadhav et al., 2007).

### 2.2. Subjects

The study participants were recruited from the Alzheimer's and Related Disorders Society of India (ARDSI), Cochin, a non-profit organization who provide medical support to dementia patients at various stages with consultation, counseling and support for family members on topics such as exercise, diet and lifestyle. ARDSI also provides a daycare facility for vulnerable patients who are not safe to be at home alone. In the present study, we recruited subjects who were not receiving any allopathic treatment but were continuously monitored by an Ayurvedic practitioner and were undergoing Ayurvedic lifestyle practices for 3–6 months. The selected subjects were prepared to continue treatment with the test substances for 6 months. Those who were healthy with respect to hematological and biochemical parameters were initially screened on the basis of MMSE score and a structured diagnostic analysis by a physician. Participants with an MMSE score between 14 and 24 were selected and the final enrolment in the study was decided on the basis of discussion between the physician and neurologist. Further to ensure a uniform baseline, individuals with no previous history of medication such as acetylcholinesterase inhibitors (AChEIs) were included in the study. Detailed inclusion and exclusion criteria are given in Table 1. Participants were also provided with incentives.

**Table 1:** Inclusion and exclusion criteria for the selection of participants for the study.

**Inclusion criteria**	**Exclusion criteria**
1. Age 55–75 years (both inclusive)	1. Severe memory loss
2. Male and female subjects	2. Stroke
3. Patients with an MMSE score of < 24 and more than 14 (both are inclusive)	3. Psychiatric illness
4. Must have a caretaker to attend the study visit.	4. Subjects who are under treatment with anti-inflammatory drugs
5. Informed consent from the subject and legally acceptable representative-lar (lar can be spouse/parent/son/daughter/guardian/person having charge or any other legally acceptable representative)	5. Objection from family members or any other close relatives or legally acceptable representatives
	6. If the participation in the study causes any harm to possible benefits to the patients including insurance benefits
	7. Objection from the physician, under whom current treatment is conducted
	8. Patients who meet the clinicians' judgment are likely to be placed in a nursing home, within the next 6 months
	9. Any condition that in the opinion of the investigator does not justify the subjects' participation in the study
	10. History of medication such as acetylcholinesterase inhibitors 1 month prior to enrolment

### 2.3. Study design and randomization

The study was conducted in accordance with the clinical research guidelines of the Government of India and the Declaration of Helsinki, following the protocol approved by the registered ethics committee of Sri Rama Hospital, Bangalore, Karnataka, India (ECR/184/Indt/KA/2014) and was retrospectively registered in India's clinical trial registry at http://ctri.nic.in (CTRI/2018/03/012410). Approximately 103 volunteers aged 55–75 years were screened, of which 64 were selected. Of the 64 subjects, 48 subjects who met the inclusion/exclusion criteria were enrolled in the study. The sample size was decided based on the power calculation with a power of 80 and 5% confidence interval; taking into account a 10% dropout rate, a minimum sample size of 40 was required for the study (Röhrig et al., 2010). The study followed a randomized, double-blinded, comparative, 3-sequence, 3-arm, parallel-group design. The enrolled participants were divided into three groups: placebo, standard curcumin complex (USC), and CGM, with each group comprising 16 participants. Randomization was performed using permuted-block randomization with a block size of 4, utilizing a computer-generated allocation table available at www.randomization.com.

### 2.4. Study protocol

**Figure 1:**
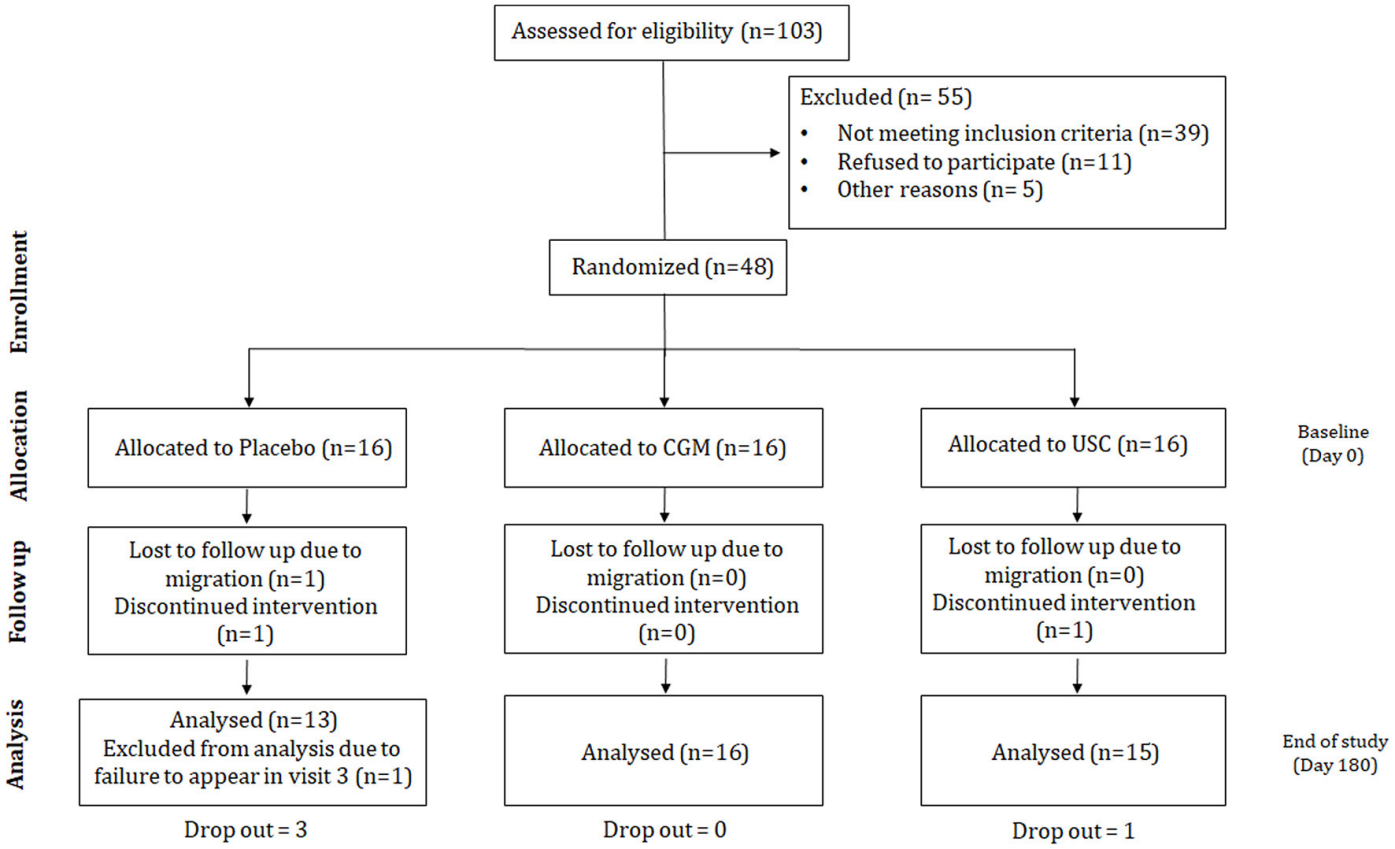
Consort diagram illustrating the experimental design of the study.

The typical protocol followed in the present study is depicted in the consort diagram (Figure 1). The total study period was 180 days (6 months). Participants were advised to follow their normal South Indian diet and exercise regularly (walking) during the course of the study period. The daily diet comprised breakfast with oats, wheat, or rice either alone or with tea/coffee; lunch made of rice and vegetables with meat or fish, and dinner including wheat, fish, and vegetables. Participants were eating an average of three eggs per week and drinking around 100 ml of milk. No other antioxidants or supplements were provided.

The protocol consists of three clinical visits on three different occasions: visit 1, screening and enrolment; visit 2, baseline/randomization (Day 0); and visit 3, end of study (EOS; Day 180). Periodical follow-ups (once every 2 weeks) were carried out via telephone in the presence of the participants' carers to ensure the adherence of the participants to the study procedures. A study diary was provided, and participants were requested to note side-effects, adverse events, any change from their routine diet, or any special events such as travel, parties, or family bereavements during the study period. Participants or carers were also advised to note the time when they consumed the supplement each day. Physical examination and laboratory tests (including red blood cell counts, packed cell volume, mean corpuscular volume, liver function tests, and renal function tests) were performed during visit 1 (screening) and visit 3 (Day 180).

Of the 103 participants screened during visit 1, 39 failed to meet the inclusion criteria, 11 refused to participate, and 5 were rejected for other reasons (difficulty in reaching the study site). The remaining 48 participants were randomly allocated to one of three groups (*n* = 16/group) and provided with three sequentially numbered airtight high-density polyethylene containers, each containing 65 sachets of CGM or USC or placebo. The baseline demographic characteristics are provided in Table 2. On visit 2 (Day 0), the subjects were instructed to consume one sachet with 220 ± 20 ml water before breakfast and dinner each day. Their adherence to the intervention instructions was checked during the follow-up visit by counting.

**Table 2:** Baseline demographic characteristics of study participants.

**Characteristics**	**Placebo (*n* = 16)**	**USC (*n* = 16)**	**CGM (*n* = 16)**
Age (years)	66.10 ± 8.5	62.78 ± 9.2	64.85 ± 7.4
Weight (kg)	74.85 ± 5.7	77.45 ± 7.8	75.12 ± 6.3
BMI (kg/m^2^)	25.17 ± 1.94	26.74 ± 1.85	25.37 ± 1.72
**Sex (** ***n*** **)**			
Males	11	10	12
Females	5	6	4
**Education**			
Below high school	2	1	3
High school and above	14	15	13

BMI, body mass index.

Values are expressed as mean ± SD.

### 2.5. Assessment of cognitive functions by MMSE score

The cognitive functions assessment was carried out using the MMSE score, which is a validated screening tool to measure overall cognitive impairment in clinical research and community settings (Folstein et al., 1975; Arevalo-Rodriguez et al., 2015). The participants were interviewed together with a responsible person from the family based on the MMSE questionnaire and were scored from 0 to 30. A participant scoring below 24 was considered as having dementia, between 21 and 24 being mild, between 10 and 21 moderate, and <10 severe onset of dementia (Pezzotti et al., 2008).

### 2.6. Assessment of locomotive function by GLFS-25

The Geriatric Locomotive Function Scale (GLFS-25) was used to assess difficulties and disabilities in activities of daily living related to locomotive organs. Seichi et al. (2012) developed this tool for the early detection of locomotive syndrome. The scale is a comprehensive self-reported measure, consisting of 25 questions referring to the preceding month. The scale includes four questions regarding pain, sixteen regarding activities of daily living, three regarding social functions, and two relating to mental health status. Each item is graded on a five-point scale, from no impairment (0) to severe impairment (4 points), and the total score is derived by the sum of all the scores (minimum = 0, maximum = 100). The total score is assumed to represent a quantitative evaluation of the difficulties and disabilities in activities of daily living related to locomotive organs. People with a score ≥16 are expected to have limitations in walking. Parameters such as slow and unstable gait and grip strength were also monitored to assess the motor functions by analyzing the intensity and speed of brisk walking as a response to question number 13, which assesses locomotive function.

### 2.7. Determination of serum biomarkers

For the analysis of serum biomarkers, blood was collected by a trained phlebotomist from the median cubital vein in a vacutainer™ collection tube. The blood samples collected from the subjects during the baseline visit (Day 0) and at the end of the study period (Day 180 ± 2 days) were used to determine the biochemical parameters and hematological parameters (RBC, Hb, PCV, MCV, and MCH). Serum was separated by centrifugation of the clotted blood sample at 2,000 × g for 10 min in a refrigerated centrifuge and used immediately for the measurement of liver function markers (AST, ALT, and ALP) and kidney function marker (serum creatinine level). Biochemical parameters related to brain functions [Aβ42 (Cat No. E-EL-H0543), tau proteins (Cat No. E-EL-H0948), BDNF (Cat No. E-EL-H0010), TNF-α (Cat No. E-EL-H0109), and IL-6 (Cat No. E-EL-H6156)] were analyzed using ELISA kits (Elabscience Biotechnology Inc., Houston, USA). The Varioskan Lux^®^ multimode reader (Thermo Fisher Scientific Inc., Marsiling, Singapore) was employed for analysis.

### 2.8. Statistical analysis

In the present study, the results of the supplementation of 400 mg of CGM for 180 days as compared to both placebo and USC were analyzed by two-way repeated measures ANOVA followed by Tukey HSD analysis. Pairwise comparison was also done using paired *t*-test. All the statistical analysis was performed using SPSS Software Version 27. The results were represented as mean ± standard deviation. A *P*-value of ≤ 0.05 was considered significant. The significance of difference is represented as a *P*-value, *P* < 0.05, *P* < 0.01, and *P* < 0.001.

## 3. Results

The baseline demographic parameters and the MMSE and GLFS-25 scores of the participants did not differ significantly (*P* > 0.05; **Tables 4A**, **B**). The biochemical and hematological parameters indicated that the participants are otherwise healthy (Table 3).

**Table 3:** Effect of placebo, USC, and CGM on clinical safety parameters.

**Parameters**	**Time**	**Groups**		
		**Placebo**	**USC**	**CGM**
Hb (g/dl)	Baseline	13.63 ± 0.65	13.94 ± 0.48	14.26 ± 0.52
	End of study	14.84 ± 0.47	14.02 ± 0.34	14.35 ± 0.56
RBC count (million/μl)	Baseline	5.78 ± 0.45	4.62 ± 0.23	4.71 ± 0.28
	End of study	5.84 ± 0.71	5.94 ± 0.25	4.44 ± 0.21
PCV (%)	Baseline	43.4 ± 2.43	44.5 ± 3.14	43.9 ± 2.73
	End of study	45.7± 2.15	46.8 ± 2.31	45.6 ± 2.55
MCV (fl/red cell)	Baseline	79.22 ± 6.35	82.59 ± 3.79	80.43 ± 4.58
	End of study	82.78 ± 2.90	84.46 ± 3.81	81.65 ± 3.69
MCH (pg/cell)	Baseline	26.07 ± 1.77	27.82 ± 1.19	25.82 ± 1.65
	End of study	27.15 ± 0.96	25.10 ± 1.23	28.45 ± 1.37
AST (IU/L)	Baseline	26.19 ± 4.61	24.62 ± 4.96	27.55 ± 3.70
	End of study	28.32 ± 4.35	27.27 ± 5.61	24.15 ± 6.75
ALT (IU/L)	Baseline	25.12 ± 5.28	26.11 ± 5.83	25.85 ± 4.33
	End of study	24.56 ± 4.88	23.78 ± 5.91	27.46 ± 3.82
ALP (IU/L)	Baseline	86.34 ± 17.10	82.46 ± 16.81	85.27 ± 10.25
	End of study	81.52 ± 13.75	86.27 ± 15.66	82.78 ± 8.78
S. Creatinine (mg/dl)	Baseline	0.96 ± 0.12	1.05 ± 0.13	0.85 ± 0.25
	End of study	0.98 ± 0.11	1.10 ± 0.14	0.93 ± 0.38

A 2 × 2 repeated measures ANOVA was employed to find out statistical significance and the obtained P-values are provided. A P-value of < 0.05 is considered statistically different.

Hb, hemoglobin; RBC, red blood cells; MCV, mean corpuscular volume; PCV, packed cell volume; MCH, mean corpuscular hemoglobin; AST, aspartate transaminase; ALT, alanine transaminase; ALP, alkaline phosphatase; S. creatinine, serum creatinine.

### 3.1. Effect on cognitive functions as monitored by MMSE scores

**Figure 2:**
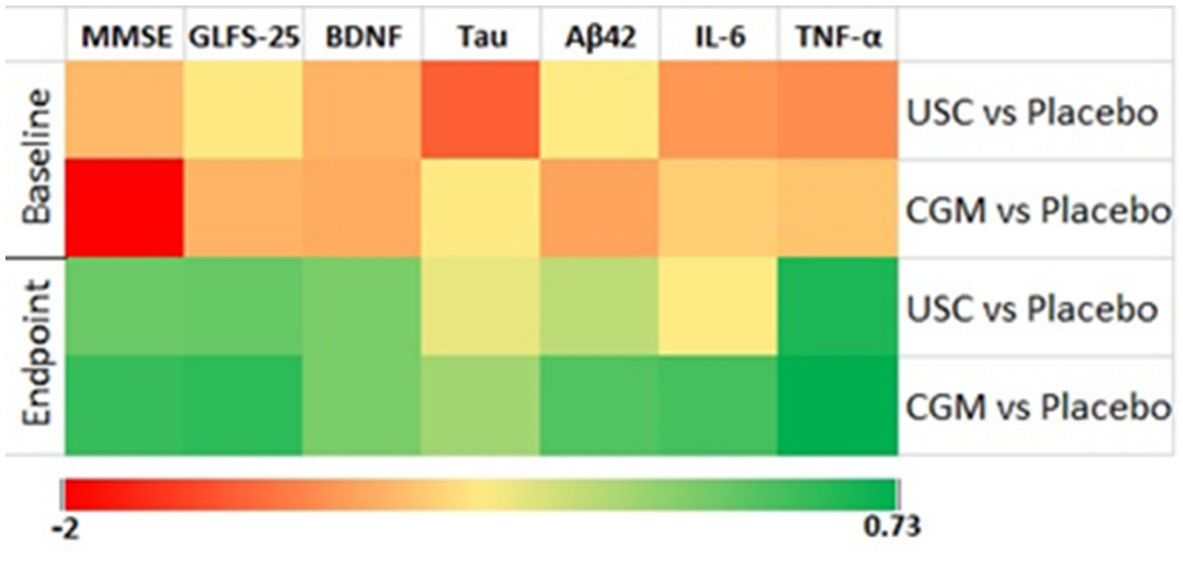
Heat map of effect size using center log-transformed data in various parameters. The groups are CGM, USC, and placebo.

The 2 × 2 repeated measures ANOVA followed by Tukey's HSD test revealed a significant main effect of CGM on the MMSE scores compared with placebo [*F*_(1, 10)_ = 11.7 and *P* = 0.007] and a significant interaction effect between the USC and CGM groups [*F*_(1, 12)_ = 23.50 and *P* = 0.001 (Tables 4A, B)]. The MMSE scores further revealed 19.09% increase for the CGM compared with the USC and 39.53% increase compared with the placebo, all of which were statistically significant (Tables 4A, B; Figure 2). Upon pairwise comparison, MMSE showed a significant (*P* < 0.001) reduction in placebo from 16.09 ± 1.86 to 13.81 ± 1.72. However, CGM supplementation significantly enhanced the MMSE score from 16.07 ± 1.81 to 19.27 ± 1.67 (19.83%; *P* < 0.001). The relative increase in the USC group was not significant (2.92%; Supplementary Table S1).

**Table 4A:** Mean and standard deviations for various parameters per interventions by Tukey's HSD test.

**Parameters**	**Time**	**Interventions**			***Post-hoc*** **test by Tukey HSD**	
		**Placebo**	**USC**	**CGM**	***F*** **-values**	***P*** **-values**
Tau protein (pg/ml)	Baseline	26.92 ± 4.37a	26.10 ± 3.83a	26.14 ± 4.27a	12.27	0.886
	End of study	31.72 ± 3.72a,b	25.86 ± 3.34b,a	24.66 ± 4.01b,b		0.001
BDNF (pg/ml)	Baseline	57.54 ± 9.25a	57.92 ± 7.55a	62.00 ± 8.53a	5.933	0.399
	End of study	55.09 ± 7.58a,a	60.00 ± 7.56b,a	66.28 ± 9.04b,b		0.005
Aβ-42 (pg/ml)	Baseline	31.70 ± 2.49a	33.31 ± 2.99a	32.06 ± 2.95a	25.37	0.898
	End of study	35.24 ± 2.72a,b	32.46 ± 2.84a,a	31.28 ± 4.48b,b		0.001
IL-6 (μg/L)	Baseline	3.40 ± 1.38a	3.64 ± 1.74a	3.70 ± 0.93a	3.332	0.848
	End of study	3.80 ± 1.72a,b	2.92 ± 1.66a,a	2.37 ± 0.50b,b		0.037
TNF-α (μg/L)	Baseline	2.04 ± 0.23a	2.09 ± 0.44a	2.12 ± 0.28a	96.01	0.804
	End of study	2.46 ± 0.36a,b	1.78 ± 0.15b,a	0.93 ± 0.29c,b		0.001
MMSE	Baseline	16.09 ± 1.86a	15.69 ± 1.70a	16.07 ± 1.81a	39.28	1.000
	End of study	13.81 ± 1.72a,b	16.15 ± 1.14b,a	19.21 ± 1.67c,b		0.001
GLFS	Baseline	17.36 ± 1.85a	16.53 ± 1.56a	16.92 ± 2.01a	40.41	0.826
	End of study	18.54 ± 1.75a,a	15.46 ± 1.45b,a	12.71 ± 1.63c,b		0.001

Significant interactions among placebo, USC and CGM groups were analyzed by Turkey HSD. The values are expressed as mean ± SD. F-value and P-value were also provided. Means not sharing subscripts differ significantly at P < 0.05 as indicated by Tukey HSD. The first superscript in “End of Study” denotes significance level of intergroup comparison by Tukey HSD *post hoc* analysis and second super script in “End of Study” denotes significance level of pairwise comparison.

**Table 4B:** Main treatment analyzed by a 2 × 2 repeated measures ANOVA for various parameters per intervention.

**Parameters**	**Time**	**Main treatment effect by 2** × **2 RM ANOVA**			
		**CGM vs. USC**		**CGM vs. Placebo**	
		***F*** **-values**	***P*** **-values**	***F*** **-values**	***P*** **-values**
Tau protein (pg/ml)	Baseline	18.31	0.001	17.04	0.002
	End of study				
BDNF (pg/ml)	Baseline	32.88	0.001	50.21	0.001
	End of study				
Aβ-42 (pg/ml)	Baseline	280.4	0.001	16.75	0.002
	End of study				
IL-6 (μg/L)	Baseline	0.549	0.473	1.727	0.218
	End of study				
TNF-α (μg/L)	Baseline	39.55	0.001	44.58	0.001
	End of study				
MMSE	Baseline	23.50	0.001	11.70	0.007
	End of study				
GLFS	Baseline	2.924	0.113	59.95	0.001
	End of study				

Main treatment effect analyzed by a 2 × 2 Repeated measures ANOVA between the placebo, CGM, and USC groups using Tukey's HSD test. F-values and P-values were provided.

### 3.2. Effect on locomotive functions as revealed by GLFS-25 scores

Supplementation with CGM showed a main effect in locomotion compared to both placebo [*F*_(1, 10)_ = 59.95 and *P* = 0.001] and USC [*F*_(1, 12)_ = 2.92, *P* = 0.113] when the GLF-25 scores were analyzed by two-way repeated measures ANOVA followed by Tukey's HSD test (Tables 4A, B). The observed reduction in the GLFS-25 score for the CGM was 31.39% compared to the placebo and 19.84% compared to the USC (Tables 4A, B; Figure 2). Pairwise comparison of the GLFS-25 scores showed no significant change in the placebo (*P* = 0.061; 6.79%; 17.36 ± 1.85 to 18.54 ± 1.75) and the USC (*P* = 0.059; 2.45%; 16.53 ± 1.56 to 15.46 ± 1.45). However, the CGM group significantly reduced (*P* < 0.001) the GLFS score from 16.92 ± 2.01 to 12.71 ± 1.63 with a relative reduction percentage of 25.69, compared to the baseline (Supplementary Table S1).

### 3.3. Effect on the biomarkers of Alzheimer's disease

#### 3.3.1. Amyloid peptide Aβ42

A significant reduction of Aβ42 was observed in the CGM group [*F*_(1, 10)_ = 16.75, *P* = 0.002] compared to the placebo and USC groups [*F*_(1, 12)_ = 280.47, *P* < 0.001; Tables 4A, B] in a two-way repeated measures ANOVA. The CGM group showed 23.26% reduction in Aβ42 when compared to the placebo and 16.02% reduction compared to the USC (Tables 4A, B; Supplementary Figures S1a, S2). No significant reduction was observed in the USC compared to the placebo (Tables 4A, B; Supplementary Figures S1a, S2). Pairwise comparison of Aβ42 further revealed 13.16% (*P* = 0.019) and 2.68% (*P* = 0.064) reduction in the CGM and USC groups, respectively, whereas the placebo group showed 11.16% (*P* = 0.001) increase (Supplementary Table S1) compared to baseline.

#### 3.3.2. Tau protein

There was a significant main effect on tau protein in the CGM treatment compared with the placebo [*F*_(1, 10)_ = 17.04, *P* = 0.002] and USC [*F*_(1, 12)_ = 18.31; *P* = 0.001; Tables 4A, B]. At the end of the study, there was a 22.79% reduction in the CGM when compared to the placebo (Tables 4A, B; Supplementary Figures S1b, S2). An intragroup comparison showed a significant reduction (*P* = 0.016) in the tau protein level in the CGM group (5.4%; from 26.14 ± 4.27 to 24.66 ± 4.01), whereas the placebo showed a significant increase (*P* = 0.05; 17.83%; 26.92 ± 4.37 to 31.72 ± 3.72 pg/ml) and the USC group showed a non-significant reduction (0.79%) compared to baseline values (Supplementary Table S1).

#### 3.3.3. BDNF

Overall, there was a significant main effect on BDNF levels for the CGM compared to both the placebo [*F*_(1, 10)_ = 50.21, *P* = 0.001] and USC [*F*_(1, 12)_ = 32.88, *P* = 0.001] groups (Tables 4A, B; Figure 2). Pairwise comparison also showed a significant increase (*P* = 0.029) for BDNF in the CGM group (from 62.00 ± 8.53 to 66.28 ± 9.04 pg/ml; ~7% increase); however, a 5.21% reduction in BDNF was observed in the placebo, which is significant (*P* = 0.001; from 57.54 ± 9.25 to 55.09 ± 7.58; Supplementary Figure S1c; Supplementary Table S1) and the USC group showed no significant change compared to baseline.

### 3.4. Effect on inflammatory markers

The two-way repeated measures ANOVA showed that CGM supplementation has a main effect on the inflammatory markers IL-6 and TNF-α [*F*_(1, 10)_ = 1.727, *P* = 0.218 for IL-6 and *F*_(1, 10)_ = 44.58, *P* = 0.001 for TNF-α] compared to the placebo. However, when compared to USC, CGM showed no significant change in IL-6 [*F*_(1, 12)_ = 0.549, *P* = 0.700 for IL-6] but significant change in TNF-α [*F*_(1, 12)_ = 39.55, *P* = 0.001; Tables 4A, B; Figure 2]. An intergroup comparison showed 51.23% reduction in IL-6 for the CGM when compared to the USC and 61.8% compared to the placebo. For TNFα, the relative reduction with respect to placebo was 31.3% and that with respect to USC was 42.01% (Tables 4A, B; Figure 2).

Pairwise comparison showed 54.12% decrease in IL-6 for CGM (*P* < 0.001) and 15.11% reduction for USC (*P* > 0.05; Supplementary Figure S2a). However, placebo showed 30.16% increase in IL-6 compared to the baseline, which was statistically significant (Supplementary Table S1). The corresponding difference for TNFα from the baseline was 54.83% (*P* < 0.003), 16.33% (*P* < 0.073), and 20.58% (*P* < 0.001) for the CGM, USC, and placebo groups, respectively, as shown in Supplementary Figure S2b (Supplementary Table S1).

### 3.5. Adverse events

No adverse events were reported in the placebo, USC, or CGM groups during the study period.

## 4. Discussion

In the current double-blinded, placebo-controlled, parallel-group, comparative study, we investigated the relative effect of a bioavailable form of curcumin (CGM) with that of unformulated standard curcumin complex with 95% purity (USC) on subjects having moderate dementia due to AD. The MMSE scores and MRI scan are well-established diagnostic tools employed in the screening of subjects to confirm the stage of dementia and the onset of AD. Previous studies with CGM have shown its enhanced bioavailability of free curcuminoids, and improved BBB permeability and brain pharmacokinetics (Krishnakumar et al., 2015; Kumar et al., 2016; Khanna et al., 2020; Kannan et al., 2021). In the current study, we investigated the relative changes in cognitive and locomotive functions and serum markers, including BDNF, tau protein, Aβ42, IL-6, and TNF-α, during the study period of 6 months. The results were promising, with considerable changes from the baseline values of the parameters tested. These results suggest that CGM may be of significant benefit in slowing down the progress of dementia and in further ameliorating the symptoms compared to the placebo. Despite previous clinical studies of curcumin and its bioavailable formulations on healthy older subjects (Gagliardi et al., 2020), the novelty of the present study lies in its comparative design with unformulated standard curcumin complex to quantify the relative influence of brain bioavailability of curcumin on Alzheimer's dementia. Moreover, the present study used the curcumin supplementation as a drink (in sachets) for the first time, which made its intake more convenient for Alzheimer's patients who have difficulty swallowing tablets and capsules.

Numerous preclinical studies have revealed the plausible potential of curcumin as a promising natural molecule for the maintenance of brain health functions (Gagliardi et al., 2020). Molecular docking and *in vitro* studies have shown that curcumin can bind to Aβ plaques and reduce Aβ aggregation by binding with β-amyloid peptide, preventing oligomer aggregation and fibril formation (Yang et al., 2005). Curcumin has also been shown to reduce tau proteins and to improve tau-mediated neuronal dysfunction and neurite abnormalities independent of insoluble tau aggregates (Miyasaka et al., 2016; lo Cascio et al., 2019). However, poor oral bioavailability, rapid biotransformation to inactive metabolites, and rapid elimination from systemic circulation have limited the therapeutic potential of curcumin in clinical settings (Liu et al., 2018; Mehrabadi et al., 2021). Table 5 shows previous clinical trials that have used curcumin and its bioavailable formulations on healthy older adults and Alzheimer's subjects. Though recent studies on healthy older adults with a free curcumin delivery form have reported cognitive improvements compared to placebo, other studies could not conclude the positive impact even when subjects were treated with 2,000 to 4,000 mg/day for 6–18 months (Table 5).

**Table 5:** Previously reported clinical studies of curcumin formulations in dementia and Alzheimer's disease.

**Study material and references**	**No. of subjects**	**Study design**	**Population**	**Dosage, curcumin content and study duration**	**Results**			
					**Placebo**		**Treated**	
					**Non-clinical parameters**	**Clinical parameters**	**Non-clinical parameters**	**Clinical parameters**
Curcumin (Baum et al., 2008)	N−27 P−8 T1−8 T2−11	DB PC RCT PGD	Alzheimer patients	1,000 mg/day 4,000 mg/day 6 months	MMSE−8.44% ↑	Aβ40−13.33% ↓ Isoprostanes—no significant change	*Results of 4,000 mg given in brackets* MMSE−3.89 % ↓ (4.48 % ↑)	*Results of 1,000 mg not provided in the article* Aβ40−25% ↑ Isoprostanes—no significant change
Longvida^®^ (Cox et al., 2015)	N−60 P−30 T−30	DB PC RCT PGD	Healthy elderly	400 mg/day 1 month	Computerized Mental Performance Assessment System-3%↑	Nil	Computerized Mental Performance Assessment System-17%↑	Nil
Longvida^®^ (Cox et al., 2020)	N−89 P−43 T−46	DB PC RCT PGD	Healthy elderly	400 mg/day 3 months	PSS−0.06% ↓ GHQ−0.13% ↑ PSQI−0.49% ↑	BDNF−1.75% ↓ Aβ42−4.17% ↓ IL-6−2.2% ↑ TNFα−6.21% ↓	PSS−3.41% ↑ GHQ−1.06% ↓ PSQI−5.48% ↑	BDNF−3.38% ↓ Aβ42−3.15% ↑ IL-6−5.32% ↓ TNFα−5% ↓
Longvida^®^ (Disilvestro et al., 2012)	N−38 P−19 T−19	PC RCT	Healthy middle- aged	400 mg/day 1 month	Nil	Plasma β-amyloid protein concentration- 4.54% ↓	Nil	Plasma β-amyloid protein concentration- 7.41% ↓
Curcumin C3 complex (Ringman et al., 2012)	N−30 P−10 T1−10 T2−10	DB PC RCT PGD	Mild to moderate AD	2,000 mg/day 6 months 4,000 mg/day 12 months	MMSE−2.15% ↓ ADAS-Cog−12.08% ↑ NPI−21.17% ↑ ADCS-ADL−9.14% ↓	Aβ42−9.4% ↑ Aβ40−5.89 % ↓ Tau−3.42% ↑	MMSE−8.41 (12.71) % ↓ ADAS-Cog−30.14 (16.06) % ↑ NPI−23.59 ↑ (41.02 ↓) % ADCS-ADL−11.94 (7.86) % ↓*Results of 4,000 mg for 12 months given in brackets*	Aβ42−0 (3.04) % ↑ Aβ40−5.55 (2.44) % ↓ Tau−0.1 (2.26) % ↑*Results of 4,000 mg for 12 months given in brackets*
Longvida^®^ (Scholey et al., 2019)	N−8	DB PC PGD	Healthy elderly	400 mg/day 3 months	Learning probe−47 % ↑ Fatigue inertia−2.5 % ↑	Nil	Learning probe−53 % ↑ Fatigue inertia−2.1 % ↓	Nil
Theracurmin™ (Small et al., 2018)	N−40 P−19 T−21	DB PC RCT PGD	Non-demented elderly	2,000 mg/day 18 months	PET Scan—↑ FDDNP binding	Nil	PET Scan-no significant change in FDDNP binding	Nil
Biocurcumax™ (Rainey-Smith et al., 2016)	N−160 P−80 T−80	DB PC RCT	Healthy elderly	1,500 mg/day 12 months	SF-36 Physical−5.65% ↓ Mental−2.71% ↓ DASS Depression−15.8% ↓ Anxiety−0.3% ↓ Stress−7.17% ↓ PRMQ−7.67% ↓	Nil	SF-36 Physical−2.31% ↓ Mental−1.56% ↓ DASS Depression−17.88% ↓ Anxiety−16.19% ↓ Stress−9.39% ↓ PRMQ−3.53% ↓	Nil
Theracurmin™ (Wynn et al., 2018)	N−36 P−19 T−17	DB PC RCT	Schizophrenia patients	360 mg/day 2 months	Nil	BDNF- 23.34% ↓	Nil	BDNF−22.07% ↑
Current study CGM-curcumin (CurQfen^®^) and Unformulated Standard curcumin (USC)	N−48 P−11 T1−14 T2−13	DB PC PGD RCT	Moderate dementia	400 mg/day 6 months	MMSE−14.17% ↓ GLFS-25−6.79% ↑	BDNF−5.21% ↓ Tau−17.83% ↑ Aβ42−11.16% ↑ IL-6−30.16% ↑ TNF-α−20.58% ↑	*Results of the USC group provided in brackets* MMSE−19.76 (2.92) % ↑ GLFS-25−25.17 (2.82) % ↓	*Results of the USC group provided in brackets* BDNF−7.15 (3.7) % ↑ Tau−5.4 (0.79) % ↓ Aβ42−13.16 (2.68) % ↓ IL-6−54.12 (15.11) % ↓ TNF-α−55 (16.33) % ↓

RCT, Randomized clinical trial; OP, open label; DB, double-blind; AC, active-controlled; PC, placebo-controlled; PGD, parallel group design; N, number of subject; P, placebo; T, treated; PRMQ, prospective and retrospective memory questionnaire; DASS, Depression Anxiety Stress Scale; CFS, chalder fatigue scale; GHQ, general health questionnaire; PSQI, Pittsburgh Sleep Quality Index; ADAS Cog, Alzheimer's Disease Assessment Scale-Cognitive Subscale, NPI, Neuropsychiatric Inventory, ADCS-ADL, Alzheimer's Disease Cooperative Study-Activities of Daily Living.

One of the major drawbacks of previous clinical studies using bioavailable formulations of curcumin was the absence of a comparative arm of unformulated standard curcumin complex so that the relative clinical efficacy brought about by the enhanced bioavailability was not clear in these studies. The positive effects observed were therefore very often blindly attributed to bioavailability. Further, most of the previous studies did not investigate the relevant biomarkers to better understand the molecular basis of these curcumin forms.

The results of the present study—that USC supplementation did not bring about significant improvement in MMSE and GLFS-25 scores—indicate its lack of beneficial effects, as reported in previous studies (Seddon et al., 2019). However, the improvement in cognitive and locomotive functions of CGM-treated subjects was clear from both the intragroup and intergroup analysis, indicating its positive effect in patients with moderate Alzheimer's dementia.

Various studies have already shown that cognition and motor functions are interrelated processes where the decline in motor function precedes or coincides with cognitive decline. Therefore, a decline in motor function, such as unusual walking patterns or unstable gait and posture, can be considered a clinical indication for dementia (Kueper et al., 2017). While the MMSE score is a widely used diagnostic tool for monitoring cognitive functions, GFLS-25 is a well-validated questionnaire for locomotive functions (Seichi et al., 2012; Myrberg et al., 2020). An MMSE score of <24 is considered as positive for dementia in Alzheimer's patients and values of <16 is considered to be severe (Pezzotti et al., 2008). Since the subjects enrolled in the present study had MMSE scores of >16, corresponding to moderate dementia due to the onset of Alzheimer's disease, the results observed primarily indicate the potential of CGM.

A non-significant variation (*P* > 0.05) in tau protein and Aβ42 level was observed in the USC group, compared to the significant reduction observed for CGM, upon both intergroup and intragroup comparison. Most of the previous studies have not reported variations in these markers, since they did not observe any significant change when subjects were treated with both the formulated and unformulated standard curcumin (Table 5). However, many preclinical studies have reported the positive effect of curcumin on these markers (Reddy et al., 2018; da Costa et al., 2019). The better influence of CGM can be attributed to its better free curcumin delivery and improved BBB permeability, as reported earlier (Krishnakumar et al., 2015; Khanna et al., 2020; Kannan et al., 2021). It is already known that the “free” unconjugated form of curcumin is a BBB-permeable molecule (Kumar et al., 2016; Cas and Ghidoni, 2019) and can inhibit β-sheet formation and bind with the fibrillar tau proteins to suppress soluble tau dimer formation *in vitro* (Rane et al., 2017; lo Cascio et al., 2019). Pathological hallmarks of AD include the deposition of amyloid β (Aβ) aggregates and the presence of neurofibrillary tangles of hyperphosphorylated tau proteins in brain tissues, leading to neuronal atrophy and death through excitotoxicity, neuroinflammation, defective calcium homeostasis, oxidative stress and energy depletion (lo Cascio et al., 2019; Silva et al., 2019). Since tau oligomers play an essential role in the misfolding of amyloid proteins and hence in the onset and propagation of AD pathology (Weller and Budson, 2018; lo Cascio et al., 2019), considerable efforts are also going into the development of therapeutics that can target tau oligomers (Gerson and Kayed, 2016).

We have also measured BDNF, a prominent neurotrophin present in the central nervous system that plays a pivotal role in synaptic plasticity and neuronal survival (Diniz and Teixeira, 2011; Jiao et al., 2016). BDNF is vital in maintaining cortical neurons of the entorhinal cortex in adults whose dysfunction results in initial short-term memory loss in AD patients (Nagahara et al., 2009; Giuffrida et al., 2018). Evidence suggests that the altered expression of BDNF and its polymorphism in the brain is closely associated with the pathogenesis of AD, with reduced BDNF levels in serum creatinine levels and CSF (Diniz and Teixeira, 2011; Jiao et al., 2016). The reduction in BDNF expression affects cognitive functions, affects learning and memory, and induces behavioral changes (Sarraf et al., 2019). Enhancing BDNF expression could thus be a strategy to prevent the loss of neurons and hence to slow down the cognitive decline in neurodegenerative conditions (Bathina and Das, 2015; Miranda et al., 2019). In the present study, supplementation with USC caused a non-significant enhancement in BDNF level, whereas the CGM group provided significant enhancement upon both intragroup (7%) and intergroup comparisons (18.14% compared with placebo) and 10.1% compared to the USC group, indicating the beneficial effect of CGM. The enhancement observed with CGM has been found to be in agreement with the previous reports (Table 5) (Wynn et al., 2018). In a randomized control trial on 36 schizophrenia subjects, supplementation with 360 mg/day of a bioavailable formulation for 8 weeks reported 22% improvement in BDNF (Wynn et al., 2018). In another study, 6-week supplementation of curcumin (500 mg/day) with iron has been reported to cause 35% improvement in BDNF (Lorinczova et al., 2020), despite the previous reports that curcumin intake in mice caused chelation of iron *in vivo* and developed significant anemia (Chin et al., 2014). Fanaei et al. (2016) reported a 40% increase in serum BDNF level upon unformulated standard curcumin supplementation at a dose of 200 mg/day for 12 weeks to women with premenstrual syndrome (Table 5).

Mounting evidence from recent studies suggests that the levels of various peripheral inflammatory markers are associated with the risk of dementia (Contreras et al., 2021; Oviedo et al., 2021; Custodero et al., 2022). The presence of damaged neurons, Aβ-aggregates, and neurofibrillary tangles stimulate the glial cells and produce proinflammatory cytokines, such as IL-1β, IL-6, and TNF-α, reactive oxygen species, and acute phase reactants, leading to chronic inflammation (Akiyama et al., 2000). The build-up of these inflammatory species significantly exacerbates the pathogenesis of AD. Inhibiting TNF-α has been identified as a promising strategy for neuroprotection and has recently been identified to ameliorate cognitive function in rodent models of AD (Decourt et al., 2017). Previous studies have shown that CGM has significant anti-inflammatory activity and prevents the synthesis of proinflammatory cytokines and generation of reactive oxygen species in various *in vivo* neurotoxic models (Mohan et al., 2019; Sheethal et al., 2020; Sunny et al., 2022). In the current study, supplementation with USC provided only a non-significant reduction for both IL-6 and TNF-α, while CGM showed a significant reduction. Though a recent meta-analysis has concluded the positive effect of curcumin on proinflammatory cytokines, the relative variations reported in different studies have resulted in diverse outcomes (Gorabi et al., 2021).

**Figure 3:**
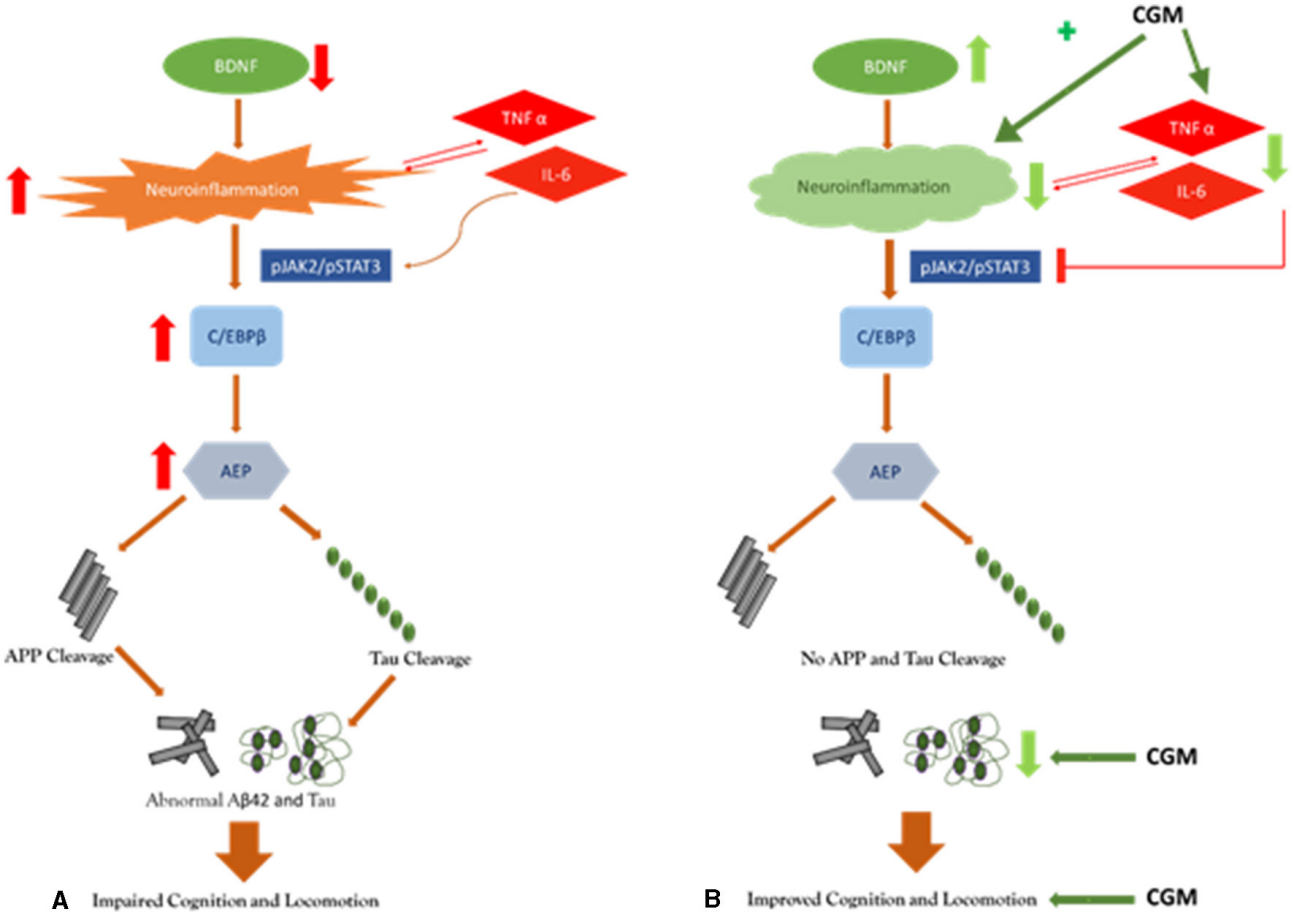
Pictorial representation of the proposed mechanism of action of CGM: **(A)** placebo, and **(B)** CGM.

Overall, from the results obtained, we hypothesize a multitargeted mode of action of CGM in the improvement of cognitive and locomotive function. CGM is primarily functioning in its effects on BDNF and decreasing inflammation as evidenced by a decrease in the cytokine, Aβ42, and tau levels (Figure 3). Wang et al. (2019) have demonstrated that the decrease in BDNF as in AD increases the level of proinflammatory cytokines. These cytokines further activate the JAK2/STAT3 pathway resulting in the upregulation of C/EBPβ/AEP signaling, leading to the cleavage of amyloid β precursor protein (APP) and tau, which ultimately leads to cognitive and locomotive impairments. Moreover, curcumin has shown to be a modulator of the JAK2/STAT3 pathway by inhibiting phosphorylation (Porro et al., 2019).

The observed improvements in the CGM group were at par with some of the previous reports on other bioavailable formulations of curcumin. However, the relatively low dosage, administration of curcumin as a beverage, and the relative efficacy based on both primary and secondary outcome measures indicated the plausible functional benefits of CGM over standard curcumin complex to delay the progress of Alzheimer's disease into dementia and ameliorate cognitive and motor functions. However, the use of a single questionnaire (MMSE) as a diagnostic tool for cognitive evaluation, the lack of ATN classification for diagnosis, and the lack of measurement of plasma curcumin concentration may be considered as the limitations of the present study.

## 5. Conclusion

Despite the advances in pharmacology and medicinal chemistry, the currently available drugs for Alzheimer's disease do not alter the course of the disease or the rate of cognitive decline. This is where the importance of utilizing safe natural molecules for the both prevention and treatment becomes evident. CGM supplementation at 400 mg/day twice daily for 6 months as a powder beverage significantly delayed the progress of dementia as evidenced by the improvements in locomotive and cognitive functions of aged-population who have been characterized with moderate dementia. The comparative and parallel group study design with unformulated standard curcumin 95% complex and placebo could establish the better efficacy of CGM. While MMSE and GLFS-25 questionnaires demonstrated the significant effect of CGM to improve cognition and locomotor effects, improvements in biomarkers (BDNF, Aβ42, and tau protein) provided the molecular evidence for the observed effect. Further, CGM also provided better control over proinflammatory cytokines (IL-6 and TNF-α). Future studies on a bigger population are recommended to better understand the relevance of ‘free' curcuminoid delivery forms such as CGM in Alzheimer's dementia.

## Data availability statement

The raw data supporting the conclusions of this article will be made available by the authors, without undue reservation.

## Ethics statement

The studies involving human participants were reviewed and approved by the registered Ethics Committee of Sri Rama Hospital, Bangalore, Karnataka, India. The patients/participants provided their written informed consent to participate in this study.

## Author contributions

Conceptualization and writing—review and editing: IK. Project administration and investigation: PG, JT, and ST. Writing—original draft preparation and data analysis: SD and MM. Writing—review and editing: BP. Resources: BM. All authors have read and agreed to the published version of the manuscript.
