# Supplementary material for: Influence of CurQfen®-curcumin on cognitive impairment: a randomized, double-blinded, placebo-controlled, 3-arm, 3-sequence comparative study
Source: Front Dement. 2023 Sep 13;2:1222708. doi: 10.3389/frdem.2023.1222708 (PMC11285547; doi:10.3389/frdem.2023.1222708)
Supplement: Supplementary file 1 [file Data_Sheet_1.docx]

Supplementary Material

**Influence of CurQfen^®^ -curcumin on cognitive impairment: A randomized, double-blinded, placebo-controlled, 3-arm, 3-sequence, comparative study**

**S. Syam Das, Prasad M. Gopal, Jestin V. Thomas, Mohind C. Mohan, Siju C. Thomas, Balu P. Maliakel, I. M. Krishnakumar, Baby Chakrapani Pulikkaparambil Sasidharan***

***Correspondence:**

Baby Chakrapani PS, Ph.D.

Centre for Neuroscience,

Cochin University of Science and Technology,

India – 682022. Phone: +91-9495109908

Email: [chakrapani@cusat.ac.in](mailto:chakrapani@cusat.ac.in), [bcps80@gmail.com](mailto:bcps80@gmail.com)

**Table 1S.** *P*-values of biomarkers, and cognitive - locomotive function scores upon intergroup and intragroup comparisons when treated with placebo, USC and CGM

| **Parameters** | **Intergroup comparison** | | | | **Intragroup comparison** | | | |  |
| --- | --- | --- | --- | --- | --- | --- | --- | --- | --- |
|  | **Placebo Vs USC** | | **Placebo Vs CGM** | **CGM Vs USC** | | **Placebo** | **USC** | **CGM** | |
| **BDNF** | | 0.080 | 0.001 | 0.001 | | 0.001 | 0.450 | 0.025 | |
| **Tau** | | 0.007 | 0.006 | 0.013 | | 0.054 | 0.058 | 0.016 | |
| **Aβ42** | | 1.000 | 0.007 | 0.001 | | 0.001 | 0.064 | 0.019 | |
| **IL-6** | | 0.090 | 0.011 | 0.027 | | 0.013 | 0.055 | 0.001 | |
| **TNF-α** | | 0.002 | 0.001 | 0.005 | | 0.001 | 0.073 | 0.003 | |
| **MMSE** | | 0.001 | 0.001 | 0.001 | | 0.001 | 0.057 | 0.001 | |
| **GLFS-25** | | 0.001 | 0.001 | 0.001 | | 0.061 | 0.059 | 0.001 | |

A paired sample t-test was employed to find out statistical significance between baseline and end of study values and independent sample t-test was performed to find out significance between groups. The obtained *P*-values are provided. *P* < 0.05 and *P* < 0.001 were considered as statistically different.


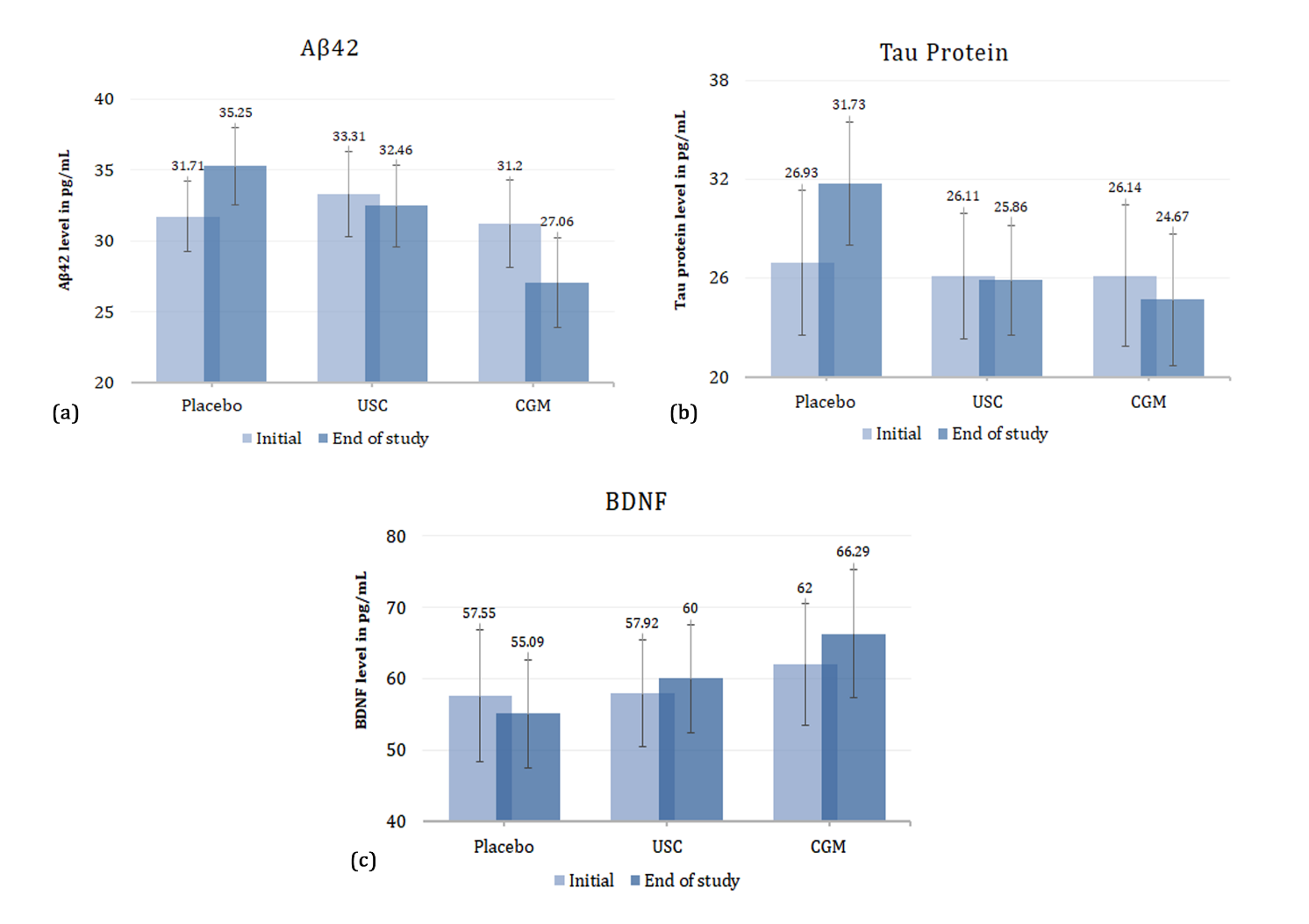


**Figure S1.** Effect of CGM on the levels of various biomarkers. Values are provided as Mean ± SD. Values having a superscript ‘*’ differ significantly with others at *P* < 0.05. **(a)** Effect of CGM on the levels of Aβ42 amyloid peptide at the baseline and at the end of study; (**b)** Effect of CGM on the levels of Tau protein at the baseline and end of study; (**c)** Effect of CGM on the levels of BDNF at the baseline and at the end of study.


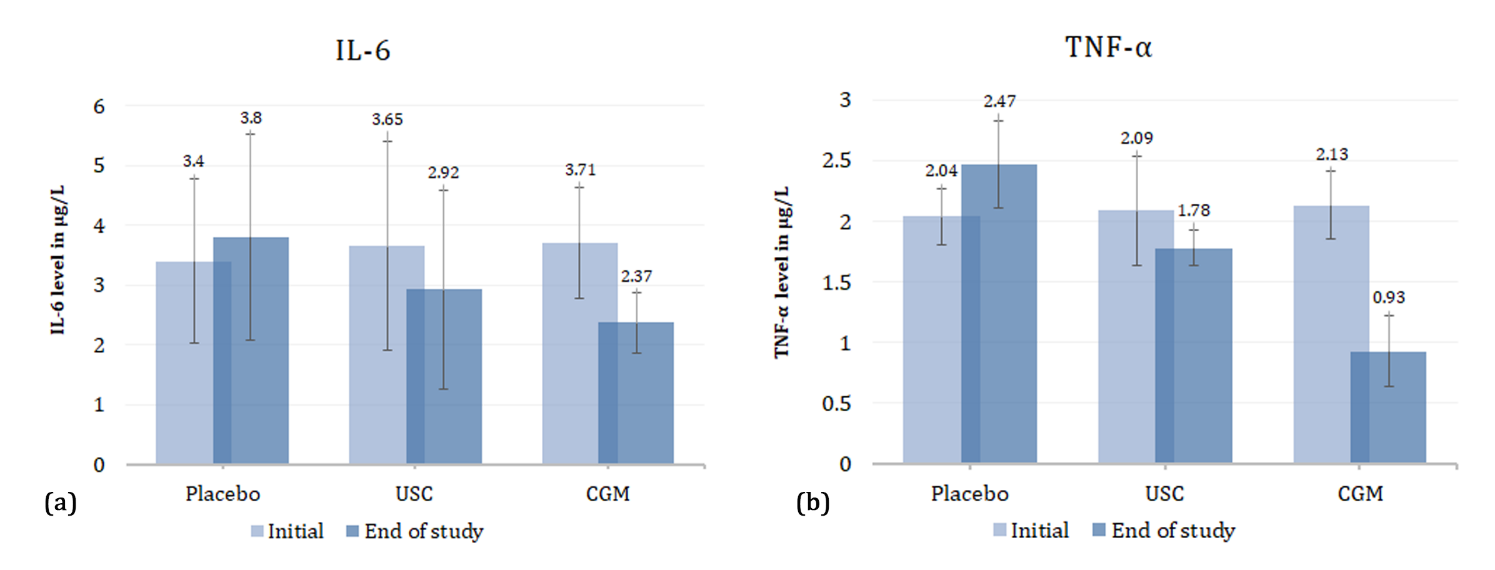


**Figure S2.** **(a)** Effect of CGM on the levels of serum IL-6 at the baseline and end of study; **(b)** Effect of CGM on the levels of serum TNF-α at the baseline and end of study. Values are provided as Mean ± SD. Values having a superscript ‘*’ differ significantly with others at *P* < 0.05.
